# Meeting of the Eastern Dental Association

**Published:** 1872-04

**Authors:** 


### Meeting of the Eastern Indiana Dental Association.

The first semi-annual meeting of the Eastern Indiana Dental Association will be held at Connersville, Indiana, May 7th, 1872.

The following subjects will be presented for discussion, viz:

1st. The supply of Lime Phosphates to the mother during the period of gestation and lactation.

2d. Mastication and Digestion.

3d. Gold Foil, soft and adhesive.

4th. The proper method of cleaning the teeth.

5th. Treatment of the exposed pulps.

6th. Miscellaneous.

W. C. Stanley, Dublin, Ind.;

J. R. Clayton, Shelbyville, Ind.;

R. F. Clayton, Shelbyville, Ind.

} Exec. Com.

